# Supplementary material for: Celebrating 40 Years of Ironman: How the Champions Perform
Source: Int J Environ Res Public Health. 2019 Mar 20;16(6):1019. doi: 10.3390/ijerph16061019 (PMC6466240; doi:10.3390/ijerph16061019)
Supplement: Supplementary file 1 [file ijerph-16-01019-s001.pdf]

Table S1. Overall times in the Ironman World Championship from 1983 to 2018 for women and men.

		MEN		WOMEN	
YEAR	POSITION	Total Race Time (s)	Total Race Time (hr:min:sec)	Total Race Time (s)	Total Race Time (hr:min:sec)
	1º🏆	**32640**	09:04:00	**38520**	10:42:00
**1983**	2º	**32700**	09:05:00	**38880**	10:48:00
	3º	**33600**	09:20:00	**39600**	11:00:00
	1º🏆	**32060**	08:54:20	**32060**	08:54:20
**1984**	2º	**33525**	09:18:45	**33523**	09:18:43
	3º	**33835**	09:23:55	**33835**	09:23:55
	1º🏆	**31854**	08:50:54	**37551**	10:25:51
**1985**	2º	**33400**	09:16:40	**37614**	10:26:54
	3º	**33992**	09:26:32	**37063**	10:17:43
	1º🏆	**30480**	08:28:00	**35340**	09:49:00
**1986**	2º	**30960**	08:36:00	**35460**	09:51:00
	3º	**32400**	09:00:00	**35940**	09:59:00
	1º🏆	**30675**	08:31:15	**33944**	09:25:44
**1987**	2º	**31519**	08:45:19	**34260**	09:31:00
	3º	**32333**	08:58:53	**33978**	09:26:18
	1º🏆	**30660**	08:31:00	**32460**	09:01:00
**1988**	2º	**30791**	08:33:11	**33133**	09:12:13
	3º	**31117**	08:38:37	**34644**	09:37:24
	1º🏆	**29355**	08:09:15	**32456**	09:00:56
**1989**	2º	**29413**	08:10:13	**33714**	09:21:54
	3º	**30736**	08:32:16	**33871**	09:24:31
	1º🏆	**30497**	08:28:17	**33222**	09:13:42
**1990**	2º	**31060**	08:37:40	**33600**	09:20:00
	3º	**31164**	08:39:24	**36033**	10:00:33
	1º🏆	**29912**	08:18:32	**32872**	09:07:52
**1991**	2º	**30274**	08:24:34	**33818**	09:23:38
	3º	**30475**	08:27:55	**34400**	09:33:20
	1º🏆	**29348**	08:09:08	**32128**	08:55:28
**1992**	2º	**29789**	08:16:29	**33700**	09:21:40
	3º	**29849**	08:17:29	**34017**	09:26:57
	1º🏆	**29265**	08:07:45	**32303**	08:58:23
**1993**	2º	**29667**	08:14:27	**32884**	09:08:04
	3º	**30013**	08:20:13	**33640**	09:20:40
	1º🏆	**30027**	08:20:27	**33614**	09:20:14
**1994**	2º	**30272**	08:24:32	**34088**	09:28:08
	3º	**30716**	08:31:56	**35010**	09:43:30
	1º🏆	**30034**	08:20:34	**33406**	09:16:46
**1995**	2º	**30179**	08:22:59	**33913**	09:25:13
	3º	**30323**	08:25:23	**34668**	09:37:48
	1º🏆	**29048**	08:04:08	**32809**	09:06:49
**1996**	2º	**29167**	08:06:07	**33079**	09:11:19
	3º	**29937**	08:18:57	**33553**	09:19:13
	1º🏆	**30781**	08:33:01	**34168**	09:29:28
**1997**	2º	**31158**	08:39:18	**34302**	09:31:42
	3º	**31119**	08:38:39	**34958**	09:42:38
	1º🏆	**30260**	08:24:20	**33652**	09:20:52
**1998**	2º	**30717**	08:31:57	**33880**	09:24:40
	3º	**30777**	08:32:57	**33974**	09:26:14
**1999**	1º🏆	**29837**	08:17:17	**33009**	09:10:09
	2º	**30174**	08:22:54	**33457**	09:17:37

	**3º**	**30342**	08:25:42	**33727**	09:22:07
	**1º🏆**	**30060**	08:21:00	**33828**	09:23:48
**2000**	**2º**	**30189**	08:23:09	**33978**	09:26:18
	**3º**	**30404**	08:26:44	**34171**	09:29:31
	**1º🏆**	**30678**	08:31:18	**33935**	09:25:35
**2001**	**2º**	**31570**	08:46:10	**34208**	09:30:08
	**3º**	**31660**	08:47:40	**34677**	09:37:57
	**1º🏆**	**30596**	08:29:56	**32704**	09:05:04
**2002**	**2º**	**30786**	08:33:06	**33105**	09:11:45
	**3º**	**30934**	08:35:34	**33446**	09:17:26
	**1º🏆**	**30155**	08:22:35	**32881**	09:08:01
**2003**	**2º**	**30767**	08:32:47	**33272**	09:14:32
	**3º**	**30951**	08:35:51	**33527**	09:18:47
	**1º🏆**	**30809**	08:33:29	**35098**	09:44:58
**2004**	**2º**	**31420**	08:43:40	**35494**	09:51:34
	**3º**	**31514**	08:45:14	**35855**	09:57:35
	**1º🏆**	**29657**	08:14:17	**32455**	09:00:55
**2005**	**2º**	**29976**	08:19:36	**32841**	09:07:21
	**3º**	**30004**	08:20:04	**32919**	09:08:39
	**1º🏆**	**29516**	08:11:56	**33226**	09:13:46
**2006**	**2º**	**29587**	08:13:07	**33522**	09:18:42
	**3º**	**29944**	08:19:04	**33657**	09:20:57
	**1º🏆**	**29734**	08:15:34	**32662**	09:04:22
**2007**	**2º**	**29944**	08:19:04	**32970**	09:09:30
	**3º**	**30090**	08:21:30	**33299**	09:14:59
	**1º🏆**	**29865**	08:17:45	**32540**	09:02:20
**2008**	**2º**	**30050**	08:20:50	**33410**	09:16:50
	**3º**	**30083**	08:21:23	**33414**	09:16:54
	**1º🏆**	**30021**	08:20:21	**31784**	08:49:44
**2009**	**2º**	**30176**	08:22:56	**32994**	09:09:54
	**3º**	**30272**	08:24:32	**33077**	09:11:17
	**1º🏆**	**29437**	08:10:37	**32064**	08:54:24
**2010**	**2º**	**29537**	08:12:17	**32467**	09:01:07
	**3º**	**29594**	08:13:14	**32730**	09:05:30
	**1º🏆**	**29036**	08:03:56	**31837**	08:50:37
**2011**	**2º**	**29351**	08:09:11	**32023**	08:53:43
	**3º**	**29467**	08:11:07	**32351**	08:59:11
	**1º🏆**	**29917**	08:18:37	**33082**	09:15:54
**2012**	**2º**	**30220**	08:23:40	**33154**	09:16:58
	**3º**	**30249**	08:24:09	**33448**	09:21:41
	**1º🏆**	**29549**	08:12:29	**31668**	08:52:14
**2013**	**2º**	**29719**	08:15:19	**31991**	08:57:28
	**3º**	**29964**	08:19:24	**32110**	09:03:35
	**1º🏆**	**29658**	08:14:18	**32188**	09:00:55
**2014**	**2º**	**29963**	08:19:23	**32308**	09:02:57
	**3º**	**30032**	08:20:32	**32403**	09:04:23
	**1º🏆**	**29680**	08:14:40	**32017**	08:57:57
**2015**	**2º**	**29863**	08:17:43	**32782**	09:10:59
	**3º**	**29930**	08:18:50	**33003**	09:14:52
	**1º🏆**	**29190**	08:06:30	**31327**	08:46:46
**2016**	**2º**	**29402**	08:10:02	**32758**	09:10:30
	**3º**	**29474**	08:11:14	**32835**	09:11:32
	**1º🏆**	**28900**	08:01:40	**31582**	08:50:47
**2017**	**2º**	**29047**	08:04:07	**32116**	08:59:38
	**3º**	**29231**	08:07:11	**32194**	09:01:38
**2018**	**1º🏆**	**28359**	07:52:39	**30039**	08:26:18

**2º**	**28601**	07:56:41	**30734**	08:36:34
**3º**	**28869**	08:01:09	**31046**	08:41:58
